# Differences in Growth Responses to Climate of Three Conifer Species in Lugu Lake of Northwestern Yunnan, Southwestern China

**DOI:** 10.3390/plants14162508

**Published:** 2025-08-12

**Authors:** Tao Yan, Yaoyao Kang, Siyu Xie, Chun Tao, Lianxiang Li, Xuefen Li, Qiong Wang, Yun Zhang

**Affiliations:** 1 College of Ecology and Environment (College of Wetlands), Southwest Forestry University, Kunming 650224, China; y_taoisme@163.com (T.Y.); xiesy_d@163.com (S.X.); 15154840844@163.com (C.T.); 2 Beijing Forestry and Parks Planning and Resource Monitoring Center (Beijing Forestry Carbon and International Cooperation Affairs Center), Beijing 101118, China; kyy0922@163.com; 3 Lugu Lake Provincial Nature Reserve Management and Protection Bureau, Lijiang 674300, China; 13988821047@163.com (L.L.); 18569802867@163.com (X.L.); yxling8888@163.com (Q.W.)

**Keywords:** climate change, dendrochronology, high mountain, radial growth

## Abstract

Responses of tree radial growth to climate are usually species-specific. Northwestern Yunnan has become a hotspot for the study of dendrochronology due to its sensitivity to climate change and the relative integrity of vegetation preservation. In this paper, we take three dominant conifers—*Pinus armandii*, *Pinus yunnanensis* and *Picea likiangensis*—as the research objects and analyze their tree-ring width chronologies in order to reveal the main climate factors affecting tree growth in northwestern Yunnan and to evaluate species-specific variation in climate response. The results showed that the radial growth of the three tree species was co-regulated by temperature and precipitation but that the growth response patterns were varied. Specifically: (1) The radial growth of the three species of conifers was significantly and negatively correlated with the July average maximum temperature (*T_max_*) and the October Palmer Drought Severity Index (PDSI) in the current year. (2) Current May precipitation significantly promoted *P. armandii* growth and inhibited *P. likiangensis* growth, and a wet July was beneficial for both *P. yunnanensis* and *P. likiangensis* growth, while the radial growth of *P. yunnanensis* and *P. armandii* showed a significant and positive correlation with the August *T_max_* in the current year. (3) The sliding analysis supported the results of the response function by showing stable relationships with climate factors which significantly affected tree growth. Results from redundancy analysis (RDA) and response function analysis were basically consistent, demonstrating that these two methods could complement each other in the understanding of relationships between tree radial growth and climatic factors. This study elucidates the climate–growth relationship of the main tree species in the study area and provides theoretical guidance and scientific evidence for regional forest management.

## 1. Introduction

In the context of global climate change, the impacts of extreme climate events (such as drought, excessive precipitation, and extremely high temperatures) on forest ecosystems are becoming increasingly prominent. The structure and function of forest ecosystems are facing unprecedented pressure [1]. Therefore, the study of the response of forest ecosystems to climate change has become a global research focus [2]. Trees are basic units of forest ecosystems, and their radial growth—reflected as tree-ring width—has been widely used in climate dynamic research, as tree radial growth provides a vital proxy of climate information, with characteristics of easy accessibility, accurate dating, high time resolution, and strong continuity [3,4]. Studying the response of tree radial growth to climate factors is conducive to understanding the growth dynamic of forest ecosystems and predicting the response of forest ecosystems to future climate change [5,6].

Subalpine forests, as an important ecosystem, exhibit high sensitivity to climate change [7]. Their areas typically include the distribution limit of elevations or latitudes where trees can hardly grow and are characterized by harsh environmental conditions such as low temperatures, strong winds, short growing seasons, long snow cover periods, and relatively fragile ecosystem structures [8]. Globally, substantial achievements have been made in studies on the responses of the radial growth of different subalpine tree species to climate change. For example, spring drought induced by climate warming and drying adversely affected tree growth in relatively arid regions of the central-western Hindu Kush Himalaya [9]. Similarly, drought induced by reduced precipitation was the primary cause of tree growth limitation in Central Asia, with the strongest constraints observed in *Picea crassifolia* Kom. (Qinghai spruce), *Sabina przewalskii* Kom. (Qilian juniper), and *Pinus sylvestris* L. (Scots pine), where all three species exhibited stable positive responses to the current spring–summer PDSI [10]. Significant linkages between tree-ring width and El Niño–Southern Oscillation were detected in high-elevation areas of the Central Andes in South America [11]. A study combining lake-core sediments and tree-ring data showed that subalpine forests presented strong post-fire recovery in the Rocky Mountains in North American, but fire frequency since the 20th century has exceeded natural variability [12]. In the European Alps, model projections indicated that for low-elevation trees, growth may suffer from drought, whereas high-elevation trees could thrive under warming [13]. These studies were crucial for evaluating the stability of subalpine forests under the background of climate change.

Over recent decades, the climate variation in northwestern Yunnan has aligned with global climate change, featuring a marked temperature increase, a negligible rise in precipitation, and an overall trend of warming and drying [14,15]. Subalpine forests are widely distributed across the area and consist of numerous tree species. How this dry-warming trend has influenced tree growth in this region has become an urgent research topic.

The northwestern Yunnan Plateau is located on the southeastern edge of the Qinghai–Tibet Plateau. With its complex terrain and deep-cut river valleys, it is a hotspot for biodiversity research [16]. Due to its high sensitivity to climate change [17], significant precipitation seasonality [18], and large diurnal temperature differences [19], it has now become a popular area for dendroclimatological research. In recent years, extensive dendrochronological studies have been conducted in northwestern Yunnan. A study on *Abies georgei* Orr (Georgei fir) on Haba Snow Mountain discovered that high-elevation *A. georgei* was primarily influenced by the maximum temperature in previous November and precipitation in current February, while low-elevation *A. georgei* was mainly constrained by drought in current May and facilitated by high temperatures in current October [20]. Photothermal conditions played a more critical role than moisture conditions on Baima Snow Mountain, and *Picea likiangensis* (Lijiang spruce) exhibited the strongest sensitivity to climate change among *A. georgei*, *Larix potaninii* Batalin (Chinese larch), and *P. likiangensis* [21]. Moisture conditions during late spring and early summer were the primary limiting factors affecting *Pinus yunnanensis* Franch. (Yunnan pine) radial growth on Yulong Snow Mountain [22]. Existing studies mostly focus on single tree species, and there are still significant deficiencies in the understanding of comparative responses of multiple tree species. Therefore, it is necessary to conduct research on the responses of different species to climate change, offering insights into growth pattern variations of different species in climate responses.

The primary aims of this study are to explore the relationship between the radial growth of three conifer species (*Pinus armandii* Franch. (Armand pine), *P. yunnanensis*, and *P. likiangensis*) and climate factors and to reveal the main climate factors affecting their growth in northwestern Yunnan. To achieve this, (1) we used response function analysis and RDA to investigate growth sensitivity to climate change; and (2) we detected the stability of the climate–growth relationships.

## 2. Result

### 2.1. Chronology Characteristics

The chronology statistics (Table 1) showed that the chronology length of the three species was almost the same, around 100 years, and that the mean sensitivity (MS) values were similar. For common interval analysis, all three species had quite high expressed population signal (EPS) values, exceeding the threshold value of 0.85, which suggested that chronologies could be used for the further climatic analysis. The three chronologies also had high values of parameters of the variance in first eigenvector (VFE) and the signal-to-noise ratio (SNR), indicating an effective representativeness within the substantial climatic information obtained.

### 2.2. Relationships Between Tree Radial Growth and Climatic Factors

**Figure 1:**
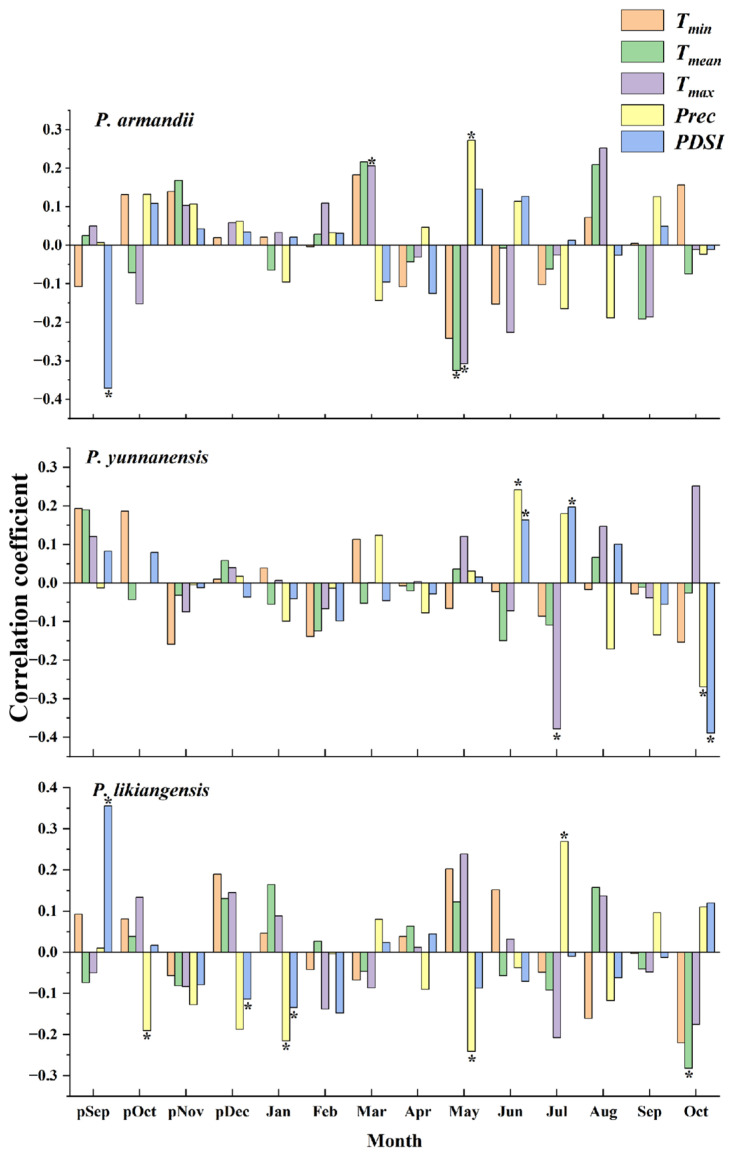
Responses of residual chronologies to monthly climate factors. p: previous year; *T_min:_* average minimum temperature; * indicates a significant correlation at *p* < 0.05.

Based on the response function analysis between the residual chronologies and climatic factors (Figure 1), the three conifer species responded quite differently to climate. Specifically, *P. armandii* showed a significant and positive correlation with the March *T_max_* and May precipitation (*Prec*) in the current year and displayed a significant and negative correlation with the PDSI in previous September, as well as with the average temperature (*T_mean_*) and *T_max_* in current May. *P. yunnanensis* exhibited a significant and positive correlation with precipitation and the PDSI in current June, as well as with the PDSI in current July, whereas its radial growth was negatively associated with the current July *T_max_* and with precipitation and the PDSI in current October. For *P. likiangensis*, significant and positive correlations were found with the PDSI in previous September and with precipitation in current July, whereas significantly negative associations were detected with precipitation in previous October, current January and May, with the PDSI in previous December and current January, and with the *T_mean_* in current October.

### 2.3. Stability of Growth Response to Climate Change

**Figure 2:**
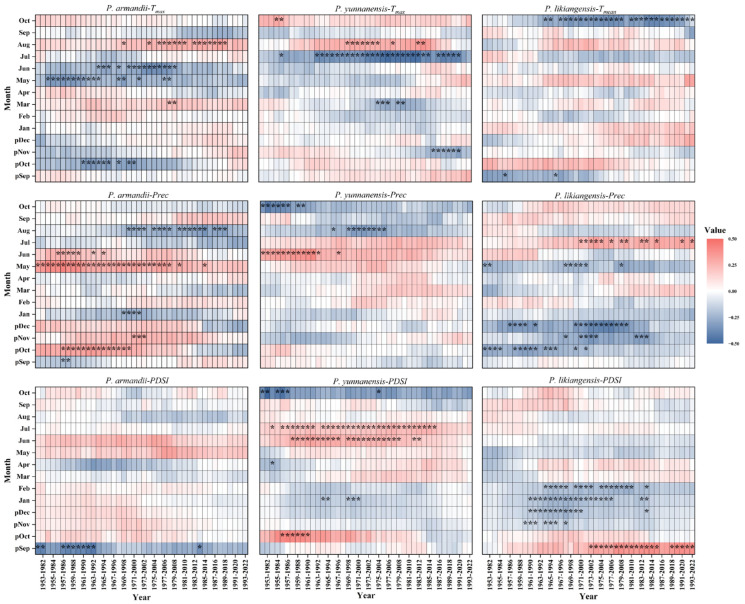
Sliding correlation analysis between residual chronologies and climate factors. * indicates a significant correlation at *p* < 0.05.

The results of 30-year moving window analysis (Figure 2) revealed interspecific differences in climatic responses. For *P. armandii*, the significance of negative correlations with the *T_max_* in current May and June mainly occurred before 2008, which corresponded to positive correlations with precipitation in current May and June. *P. yunnanensis* exhibited a gradual strengthening in the significance of its correlation with the current July *T_max_*, corresponding to the period of significant years of summer (June–July) of the PDSI. For *P. likiangensis*, the radial growth of negative associations with the *T_mean_* in current October was quite stable between 1964 and 2022. The positive impacts of July precipitation on growth were more obvious from 1971 onward by showing more significant years. Significant correlations between growth and the winter (previous November to current February) PDSI were mainly observed between 1961 and 2013.

### 2.4. Redundancy Analysis

**Figure 3:**
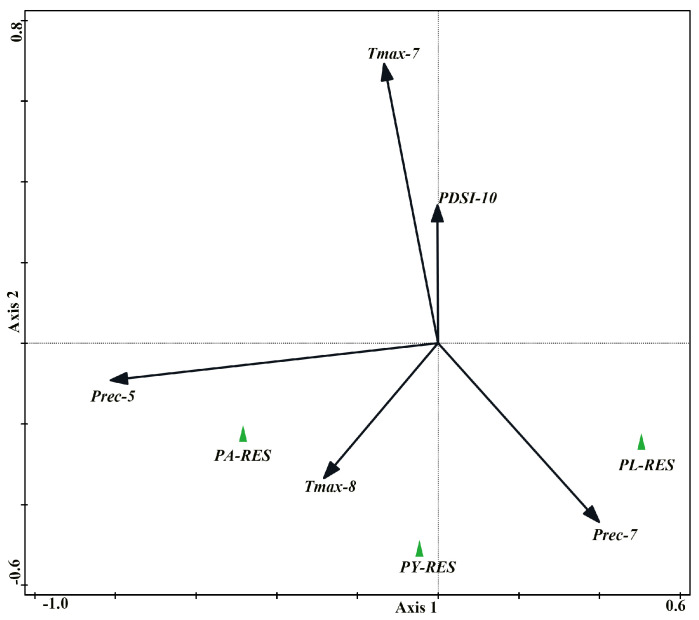
Redundancy analysis of residual chronologies with climate factors. Only significantly correlated climate factors were selected. The longer the vector of climate factors, the higher the contribution, the shorter the vertical line between the chronological point and the vector (itself or the extension line), and the higher the correlation between the two. The same direction of the two indicates a positive correlation, while the opposite is a negative correlation. PA-RES: residual chronology of *P. armandii*. PY-RES: residual chronology of *P. yunnanensis*. PL-RES: residual chronology of *P. likiangensis*.

The results of RDA (Figure 3) showed that the first and second axes collectively explained 29.95% of the variance in the response variables, with the first axis accounting for 17.03% and the second axis 12.92%. Among the 70 examined climate variables, five climate variables significantly influenced (*p* < 0.05) the radial growth of the studied species. The July *T_max_* and the October PDSI in the current year had the same effects on tree growth by showing negative associations with the three conifer species. Both current May precipitation and the August *T_max_* had positive impacts on the radial growth of *P. armandii* and *P. yunnanensis*, while these two climate variables negatively affected *P. likiangensis* growth. Current July precipitation enhanced the radial growth of *P. likiangensis* and *P. yunnanensis* and had a negative impact on *P. armandii* growth.

## 3. Discussion

### 3.1. Common Climatic Responses in Radial Growth of Three Conifer Species

Both the July *T_max_* and the October PDSI in the current year inhibited the radial growth of three conifer species, and this phenomenon was more obvious in *P. yunnanensis* than in the other two species. July is the peak period for tree growth, but high temperatures could exacerbate soil moisture evaporation and plant transpiration [23]. Furthermore, high temperatures in July disrupted the balance between photosynthesis and respiration in trees. High temperatures caused plant stomata to close, reducing carbon dioxide uptake and limiting photosynthesis [24], thereby decreasing the accumulation of photosynthates. Meanwhile, high temperatures enhanced respiration, consuming more photosynthates and resulting in insufficient energy and substances for tree growth [17]. The promoting effect of July precipitation on *P. yunnanensis* and *P. likiangensis* also illustrated the inhibitory impact of drought from another perspective. If precipitation did not increase correspondingly in July, soil moisture supply became insufficient, subjecting trees to drought stress. In July, with the onset of the rainy season, increased precipitation provides sufficient water for trees, promoting photosynthesis and growth [25]. A similar response pattern has also been observed in the Hengduan Mountains [26].

Although *P. yunnanensis* was drought tolerant, severe drought could still affect its growth. That the phenomenon of drought stress was the main factor limiting the radial growth of *P. yunnanensis* has been confirmed in the Nanpan River Basin, central Yunnan Plateau [15]. The negative impact of July temperatures on tree growth was stable for *P. yunnanensis* and *P. likiangensis* by showing significances during most of the study period, suggesting the importance of July water conditions on tree growth and confirming the reliability of correlation analyses. Because *P. armandii* demands more light compared with the other two species, during the rainy month of July, excessive precipitation led to frequent cloudy days with less solar radiation and sunlight, and this could have decreased photosynthetic efficiency and thus inhibited the growth of *P. armandii* [27]. This viewpoint has also been supported by a study of *P. armandii* in Longchiman, on the eastern edge of the Qinling Mountains [28].

**Figure 4:**
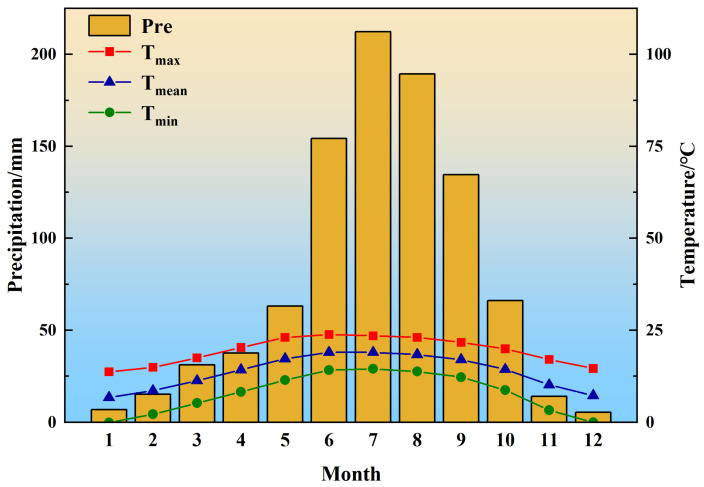
Climate data from 1950 to 2023. Scale fixed: P (mm) = 2 × T (°C).

The negative impact of the October PDSI on tree growth highlighted the importance of water conditions in the late growing season. Generally, moist conditions in the late growing season (October) were considered conducive to tree growth [29]. However, our study showed that wetter conditions inhibited tree growth; this could reflect both direct and indirect impacts of moisture conditions on tree growth. Although trees almost stop growing in October, the daily temperature remains above 5 °C (Figure 4), and the physiological activities of trees do not completely cease. Firstly, excessive precipitation could result in soil moisture saturation, thereby impairing root activity and subsequently inhibiting tree radial growth [30]. Additionally, excessive soil moisture could reduce soil oxygen availability while increasing carbon dioxide concentrations, thereby limiting root activity and the photosynthetic efficiency of trees [31]. Secondly, higher humidity means more precipitation with lower temperatures, which could reduce light intensity and could weaken tree photosynthesis, leading to decreased organic matter accumulation for growth [32].

### 3.2. Differential Climatic Responses Among Species

Trees growing in the same environment often exhibit differences in their responses to climatic factors due to variations in species biological adaptability [33]. Excessive precipitation in current May promoted the radial growth of *P. armandii* and *P. yunnanensis*—particularly for *P. armandii* which also showed many significant correlations in the sliding analysis. Conversely, excessive precipitation in May had a negative impact on the growth of *P. likiangensis*. The result of the different demands of these two species was reflected in the radial growth in spring. *P. armandii* growth exhibited a significant positive correlation with precipitation and a significant negative correlation with temperature, indicating that its growth was constrained by drought stress [34]. As temperatures increased gradually in May, thermal conditions satisfied the trees’ growth requirements, thereby rendering precipitation the primary limiting factor. The rise in temperature enhanced soil water evaporation; insufficient precipitation during this period thus induced drought stress, thereby inhibiting growth [27]. This phenomenon was also observed in other species in nearby areas [35,36]. Conversely, excessive precipitation was detrimental to *P. likiangensis* presumably due to its shallow-root character and high sensitivity to soil moisture fluctuations, which enable rapid absorption of surface soil water [37]. Excessive May precipitation was observed to induce soil anoxia and root damage, thereby compromising root growth and nutrient uptake capacity and disrupting normal physiological processes [38].

The August *T_max_* was also an important climatic factor that affected the radial growth of the three conifer species, as detected by RDA, although this relationship was not significant in the response function, suggesting that the two methods can complement each other. This influence was more obvious for *P. yunnanensis* and *P. armandii* by showing many significances in the sliding analysis, demonstrating the critical role of elevated temperatures in August in their radial growth. High temperatures during the growing season were found to facilitate photosynthetic production of organic compounds, consequently promoting the development of wide annual rings [39]. In the central region of the Hengduan Mountains, it has been reported that current August high temperatures promoted tree growth [40].

### 3.3. Species-Specific Climatic Responses

*P. likiangensis* is a shallow-rooted species which is more susceptible to low-temperature freezing damage. December and January are the coldest times in the studied area, and precipitation often accompanies lower temperatures, causing damage to the roots of the tree species and affecting the absorption of nutrients in the following year, which is not conducive to tree growth. The negative influence of winter precipitation on tree growth was also found in a subalpine forest in nearby western Sichuan [41].

The study revealed that the radial growth of *P. yunnanensis* was likely controlled by summer (June–July) moisture conditions by showing a positive correlation with precipitation and the PDSI during the period, suggesting that this species demanded more water. Elevated summer temperatures exacerbated plant transpiration and accelerated soil water evaporation [42], resulting in a water deficit that was insufficient to meet the moisture demands of photosynthesis. This water limitation subsequently restricted cambial cell division, hampered tree growth, and ultimately induced drought stress [27,43]. Conversely, adequate precipitation during this period effectively alleviated water scarcity, promoted the synthesis of photosynthetic products, and facilitated tree growth [44].

This study demonstrated that the radial growth of the three tree species (*P. armandii*, *P. yunnanensis*, and *P. likiangensis*) was jointly influenced by temperature and precipitation. Among the three species, only the radial growth of *P. likiangensis* showed a more significant correlation with climatic factors in the previous year, a phenomenon known as the lag effect [45], which had also been reported in the western Sichuan Plateau [46]. The cambial activity of spruces (including *P. likiangensis*) was initiated earlier, relying on nutrients stored from the previous year to initiate cell division, whereas the activation of cambium in pine species (e.g., *P. armandii* and *P. yunnanensis*) depended more on immediate spring climatic signals in the current year [47].

## 4. Materials and Methods

### 4.1. Study Area and Species

**Figure 5:**
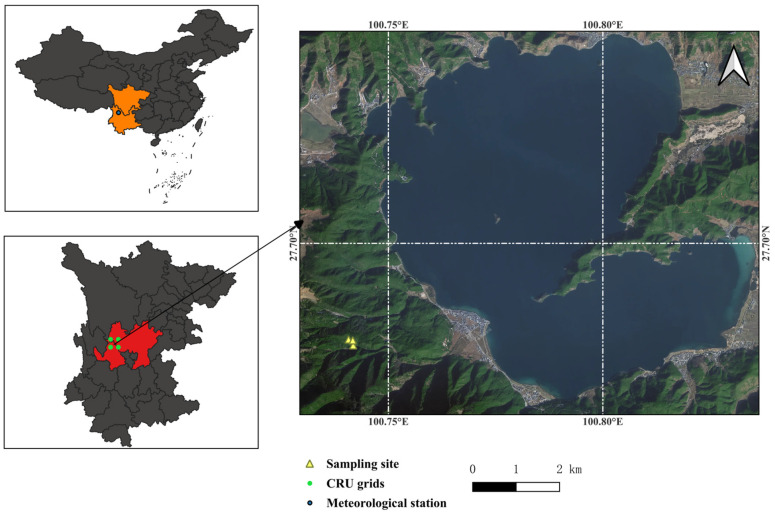
Location of the study area and sampling sites. In this figure, the orange-colored regions represent Yunnan Province and Sichuan Province; the red-colored regions represent Ninglang County and Yanyuan County.

Lugu Lake Nature Reserve (100°43′–100°50′ E, 27°37′–27°45′ N) is located at the junction of the Qinghai–Tibet Plateau and the Yunnan–Guizhou Plateau [48]. It is within Ninglang County of Yunnan Province and within Yanyuan County of Sichuan Province, belonging to the low-latitude plateau monsoon climate zone (Figure 5). It has sufficient sunlight, warm winters, cool summers, moderate precipitation, and a small annual temperature difference. Over the past 70 years, the central region of the Hengduan Mountains has experienced a significant warming trend, with a rate of 0.3 °C per decade, while precipitation has not increased substantially, resulting in a dry-warming trend [14,15]. The average altitude of the Lugu Lake Nature Reserve is 2690 m, and the highest peak is 3869 m. *P. yunnanensis* is a principal tree species in southwestern China [49] and predominantly occurs at elevations of 1500–3200 m in the study area. It exhibits a high ecological value [50] and is characterized by rapid growth, strong adaptability, and tolerance to drought and barren soils [51]. *P. armandii* serves as a fast-growing timber tree in southwestern China [52] and thrives at altitudes of 1600–3200 m [53]. It prefers cool, humid climates and deep, moist, well-drained acidic soils. This species also demonstrates cold tolerance but shows a sensitivity to aridity and high temperatures [52]. *P. likiangensis* functions as a dominant tree in the alpine forests of northwestern Yunnan, concentrating within the 2800–3500 m vertical belt [54]. It displays shade-, drought-, and cold-tolerant characteristics, with a shallow root system, featuring well-developed lateral roots and a preference for well-drained acidic soils [55]. These three conifer species are all dominant tree species in the study area.

### 4.2. Climate Data

**Figure 6:**
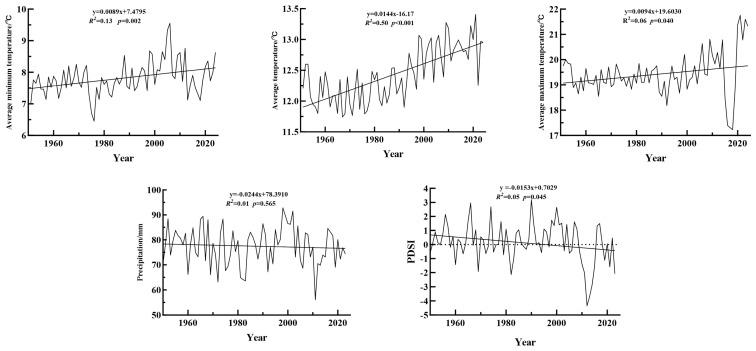
Climate trends.

For this study, the meteorological data were sourced from the National Oceanic and Atmospheric Administration (NOAA). Two meteorological factors, namely the *T_min_* and *T_max_* from 1950 to 2023, were downloaded from the NOAA Lijiang Meteorological Station (100.22° E, 26.85° N, altitude 2380 m), which is the closest station to the sampling sites. According to the meteorological data from 1950 to 2023 (Figure 4), the annual *T_mean_* is 13.6 °C. The warmest month is July with a temperature of 18.9 °C, and the coldest month is January with a temperature of 2.55 °C. The temperature shows an upward trend over time (Figure 6), with the *T_max_*, *T_mean_*, and *T_min_* in Lijiang all rising to varying degrees. Among them, the *T_max_* has been increasing at the fastest pace annually, while the *T_mean_* is rising at a relatively slower rate (Figure 6). The precipitation and the *T_mean_* data were obtained from the Climatic Research Unit (CRUTSv.4.07; https://crudata.uea.ac.uk/cru/data/hrg, accessed on 20 April 2023), with a spatial resolution of 0.5° × 0.5°. The annual total precipitation is 930 mm, and precipitation is concentrated from June to September, accounting for 74.2% of the total precipitation, with the highest precipitation in July (Figure 4). The annual total precipitation shows a decreasing trend over time, but the reduction is not significant (Figure 6). The PDSI data were retrieved from the monthly dataset compiled by the global CRU grid (https://crudata.uea.ac.uk/cru/data/drought, accessed on 20 April 2023), with a resolution of 0.5° × 0.5°. The PDSI, a comprehensive meteorological drought indicator that combines precipitation, evapotranspiration, and drought duration, is widely used in drought assessment [56]. The PDSI generally shows a declining trend (Figure 6), indicating that the climate is becoming increasingly arid.

### 4.3. Tree-Ring Sampling and Processing

**Figure 7:**
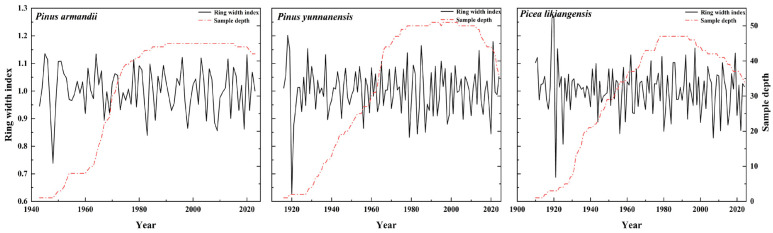
Residual chronologies and sample size of *P. armandii*, *P. likiangensis,* and *P. yunnanensis* tree-ring widths.

In March 2024, tree cores of *P*. *armandii*, *P*. *yunnanensis*, and *P*. *likiangensis* were collected in three sites of Lugu Lake Nature Reserve, with each sampled stand being respectively dominated by each studied species. The density of each stand was not very high, and the soil was primarily acidic mountain brown forest soil (Table 2). During sampling, efforts were made to select trees with relatively old ages, healthy growth, and minimal human interference. An increment borer with an inner diameter of 5.15 mm was used to drill holes at a height of 1.3 m above the ground. Two cores per tree were collected from two different directions and quickly placed into plastic tubes, which were then numbered and labeled [57]. A total of 78 trees of the three species were sampled, yielding 157 cores (Table 2). The cores were processed in the laboratory following the basic procedures for tree-ring processing [58]. The cores were fixed in specially made wooden grooves and left to air-dry, and then they were polished with sandpaper of gradually finer grits until the tree rings were clearly visible. Subsequently, the cores were visually cross-dated under a binocular microscope and scanned using an EPSON (Expression 11000XL) scanner (Seiko Epson Corporation, Suwa, Japan). The scanning parameters were set as a 24-bit full-color image type and a resolution of 2500 dpi. The scanned core images were sorted, and the tree-ring widths were measured using the software CDendro and CooRecorder ver. 7.3 with a system accuracy of 0.001 mm [59]. The cross-dating and result verification were carried out using the COFECHA program [60]. Cores with low correlations with the main sequence were removed. Finally, 77 trees and 143 cores (Table 1) were retained for the main sequence. The ARSTAN program was used to fit the data with a 67% spline function to remove the growth trends caused by the trees’ own genetic factors [61]. Standard chronologies (STD), autoregressive chronologies (ARS), and residual chronologies (RES) of *P. armandii*, *P. likiangensis*, and *P. yunnanensis* were established, and the RES (Figure 7) were generated in order to eliminate autocorrelation effects, enhance common signals, and be subsequently used for climate–growth relationship analysis.

### 4.4. Data Analysis

To avoid the influence of the “lag effect” [45], five types of analyses for assessing climate data from September in the previous year to October in the current year were selected for the correlation analysis with the tree-ring width index, aiming to explore the responses of the radial growth of three conifer species to climate change. Response function analysis was conducted by using the DendroClim2002 software [62]; this is an approach that involves extracting principal components from climatic factors before performing regression analysis, enabling a more accurate reflection of the degree to which sample data are influenced by environmental factors [63]. Sliding correlation analysis was carried out using the Evolutionary and Moving Response and Correlation module within the DendroClim2002 software (with a 30-year sliding window), allowing for a comprehensive understanding of the stability of the relationship between tree radial growth and climatic factors. Furthermore, the relationship between tree radial growth and climatic factors was further validated by using RDA of the CANOCO 4.5 software [64]. RDA, a multivariate environmental gradient analysis technique, evaluates the relationship between tree radial growth and climatic factors through regression and principal component analysis of chronologies and climatic variables [65]. The graphs were drawn by using Origin 2025 and GraphPad Prism 10.1.

## 5. Conclusions

The growth response to climate change was varied among the three studied conifer species due, to some extent, to their biological characteristics. The current July *T_max_* and the October PDSI were limiting factors that restricted the radial growth of these three conifer species. *P. armandii* was mainly controlled by spring precipitation, and *P. yunnanensis* was mainly controlled by summer moisture conditions; the growth response pattern was the most complex for *P. likiangensis* because winter moisture conditions, spring temperatures, and summer July drought had influences on its growth. These were identified as the key climatic factors affecting tree growth in the study area. Additionally, the lag effect of climate was only notably evident in *P*. *likiangensis*. In dendroclimatological research, response function analysis and RDA were found to effectively complement each other, enabling a more comprehensive understanding of the pattern governing tree radial growth. The findings of this study contribute to elucidating the primary climatic factors that affected the radial growth of three conifer species in northwestern Yunnan and provide a scientific basis for forest resource management and conservation.

## Figures and Tables

**Table 1:** Statistics of residual chronologies and common interval analysis.

Chronology	*P. armandii*	*P. yunnanensis*	*P. likiangensis*
Sample No.	25/45	26/50	26/48
Chronology/a	1937–2023	1913–2024	1906–2024
Mean sensitivity (MS)	0.09	0.08	0.07
Common interval/a	1969–2022		
Variance in first eigenvector/% (VFE)	35.45	30.80	33.18
Standard deviation	0.07	0.07	0.06
Signal-to-noise ratio (SNR)	11.26	14.67	13.60
Expressed population signal (EPS)	0.92	0.94	0.93

**Table 2:** Sampling information and general growth requirements of the three conifer species.

	*P. armandii*	*P. yunnanensis*	*P. likiangensis*
Elevation/m	3145	3210	3082
Latitude (N)	27°40′38″	27°40′34.39″	27°40′39.33″
Longitude (E)	100°44′30″	100°44′30.45″	100°44′26.6″
Number of trees/tree cores	26/53	26/52	26/52
Aspect	SW	S	SE
Slope (°)	10°	10°	6°
Elevation range	1600–3200 m	1500–3200 m	2800–3500 m
Suitable temperature	12–18 °C	15–20 °C	7–12 °C
Suitable precipitation	478–1870 mm	800–1200 mm	500–1000 mm
Distribution areas	Central China Western China Southwestern China	Southwestern China	Southwestern China

## Data Availability

The original contributions presented in the study are included in the article; further inquiries can be directed to the corresponding author.
